# The Biological Functions of Yeast and Yeast Derivatives and Their Application in Swine Production: A Review

**DOI:** 10.3390/microorganisms13071669

**Published:** 2025-07-16

**Authors:** Yuyang Fan, Chenggang Yin, Lei Xu, Rong Bai, Zixi Wei, Ge Gao, Yanpin Li, Wenjuan Sun, Xilong Li, Yu Pi

**Affiliations:** 1 Key Laboratory of Feed Biotechnology of Ministry of Agriculture and Rural Affairs, Institute of Feed Research, Chinese Academy of Agricultural Sciences, Beijing 100081, China; w18832417225@163.com (Y.F.); ycg0701@126.com (C.Y.); xlei0611@163.com (L.X.); bairongkelsi@163.com (R.B.); 13126830289@163.com (Z.W.); 82101192355@caas.cn (G.G.); liyanpin@caas.cn (Y.L.); sunwenjuan@caas.cn (W.S.); lixilong@caas.cn (X.L.); 2 Precision Livestock and Nutrition Unit, TERRA Teaching and Research Centre, Gembloux Agro-Bio Tech, University of Liège, 5030 Gembloux, Belgium

**Keywords:** yeast, yeast derivatives, bioactivity, pig, health

## Abstract

Yeast and its derivatives, including yeast extract and yeast cell wall, are well established as safe and environmentally sustainable feed additives that significantly improve animal production performance and health. Their incorporation into swine production serves as an innovative nutritional strategy aimed at improving growth performance, bolstering health status, and enhancing immune function in pigs. As a versatile microorganism, yeast generates a variety of bioactive compounds through fermentation, such as amino acids, vitamins, enzymes, and growth factors, which collectively contribute to improved growth and overall health in pigs. This review consolidates current research on the utilization of yeast and yeast derivatives in swine production, highlighting their biological functions and practical implications within the industry.

## 1. Introduction

Since the 20th century, antibiotics have been extensively used as growth promoters in intensive livestock farming systems. However, their prolonged and widespread application has led to serious concerns regarding antimicrobial resistance and drug residues, posing significant threats to both livestock product quality and public health [1]. In this context, yeast and its derivatives have emerged as promising natural alternatives, garnering considerable research attention. Over the previous decades, studies have demonstrated that yeast supplementation not only enhances feed efficiency, nutrient digestibility, and production performance, but also exhibits remarkable antimicrobial effects and mitigates the environmental impact of feed additives [2,3,4]. Yeast-derived bioactive compounds have gained widespread attention due to their positive effects on animal health. These compounds are rich in essential nutrients such as β-glucans, nucleotides, amino acids, and proteins, playing a pivotal role in maintaining physiological homeostasis. Particularly noteworthy is their demonstrated therapeutic potential in alleviating oxidative stress, inflammation, and associated diseases [5,6,7]. Numerous studies have confirmed that dietary supplementation with yeast-derived bioactive compounds significantly improves the production performance of piglets by modulating gut microbiota and enhancing intestinal morphology. For instance, the inclusion of β-glucans in weaned piglet diets has been shown to optimize gut microbiota composition and reduce diarrhea incidence, thereby improving overall growth performance [8]. Similarly, chitosan supplementation has been reported to promote piglet growth by elevating serum growth hormone levels and reinforcing small intestinal structural integrity [9].

Yeast-derived bioactive compounds exhibit multifaceted benefits, including antimicrobial activity, anti-inflammatory effects, and enhanced antioxidant capacity. β-glucans, for example, exhibit potent antimicrobial properties in pig production, with their unique structural characteristics—such as thick cell walls and small particle size—facilitating macrophage colonization and enhancing the antimicrobial functions of monocytes and neutrophils [10,11]. Additionally, dietary supplementation with mannan oligosaccharides has been shown to significantly reduce pathogenic colonization in the cecum and attenuate inflammatory responses, while chitin derivatives, particularly chitin oligosaccharides and chitosan oligosaccharides, demonstrate notable anti-inflammatory effects [12,13].

Multiple studies have demonstrated that dietary supplementation with yeast and its derivatives significantly enhances pigs’ anti-inflammatory, antioxidant, and immune functions, ultimately improving growth performance [14,15,16]. For example, under heat stress conditions, supplementation with 0.25% live yeast has been shown to reduce plasma levels of the pro-inflammatory cytokine TNF-α, thereby mitigating inflammatory responses [17]. Moreover, chromium yeast supplementation has been found to increase serum concentrations of immunoglobulin G (IgG) and immunoglobulin M (IgM), enhancing immune responses in pigs [16]. Additionally, the addition of 0.125% yeast-derived postbiotics has been found to elevate IgG and IgA levels in sow milk and IgG and IgM levels in piglet serum, further supporting immune system health [18].

This review aims to systematically summarize the applications of yeast in swine production, focusing on its effects on growth performance, feed utilization efficiency, and immune function. By synthesizing current research findings, this paper seeks to provide valuable insights and references for the development of innovative strategies in the swine industry, ultimately promoting sustainable development and productivity enhancement in the sector.

## 2. The Main Sources of Yeast in Pig Production

Yeast is a unicellular microorganism classified within the fungi kingdom and functions as a facultative anaerobe. It is rich in protein, vitamins, digestive enzymes, and various trace elements, while its cell wall contains abundant yeast polysaccharides. Due to its rapid proliferation and high nutritional value, yeast is widely utilized as a feed additive in animal production. To date, over 1500 yeast species have been identified.

Yeast species commonly used in livestock and poultry production include *Saccharomyces cerevisiae*, beer yeast, *Pichia* yeast, *Kluyveromyces fragilis*, *Saccharomyces boulardii*, *Candida* yeast, *Debaryomyces* yeast, *Ascomycete* yeast, *Metschnikowia* yeast, and *Issatchenkia* yeast. These yeasts have been extensively applied in the animal husbandry industry due to their distinct biological properties and functional benefits.

### 2.1. Saccharomyces cerevisiae

*Saccharomyces cerevisiae* (*S. cerevisiae*) is a highly versatile microorganism that has been traditionally used in food and beverage production. Beyond its conventional applications, it plays a crucial role in biotechnology, biofuel production, and bioremediation. *S. cerevisiae* possesses a flexible genome of approximately 12 megabases, comprising around 6000 genes, making it an ideal model organism for research and industrial applications [19].

As a single-celled eukaryotic microorganism, *S. cerevisiae* provides a broad spectrum of essential nutrients for living cells, including proteins, oligopeptides, amino acids, and polysaccharides, particularly glucans and mannans. Additionally, it contains lipids, B complex vitamins (excluding vitamin B12), and essential minerals such as chromium, selenium, zinc, copper, phosphorus, magnesium, calcium, iron, and manganese [20]. Research indicates that *S. cerevisiae* cells have a higher protein content compared to conventional animal- and plant-based protein feeds. Furthermore, its rapid growth and reproduction rates enable significantly greater productivity than traditional protein feed sources.

Moreover, *S. cerevisiae* protein production utilizes a diverse range of raw material sources, including industrial and agricultural by-products such as industrial wastewater, brewery waste, discarded beet pulp, fruit pomace, straw, and petroleum processing residues. The ability to convert these waste materials into raw materials and single-cell protein feed not only mitigates environmental pollution but also enhances resource efficiency by transforming waste into valuable resources [21].

### 2.2. Beer Yeast

Beer yeast is an industrial by-product generated during the beer brewing process. The solid residues formed during the brewing are rich in yeast cells, which can be dried and processed into beer yeast powder [22]. As a single-celled fungus, it is abundant in digestible proteins, beneficial microorganisms, and essential mineral elements [23]. It contains approximately 40–50% protein [24], 35–45% carbohydrates (primarily glucans and mannose), 5–10% fat (mainly unsaturated fatty acids), 10–15% fiber, and 4.5% to 8.3% nucleic acid [25].

Currently, beer yeast is widely utilized across a multitude of sectors, including fermentation, food processing, animal feed production, agriculture, bioenergy, pharmaceuticals, chemicals, and environmental conservation. As a cost-effective and nutrient-dense by-product of beer brewing, it represents an ideal and economical resource for multiple applications [26]. Beer yeast serves as an excellent nitrogen source in animal nutrition, offering significant nutritional benefits. The metabolic byproducts of this yeast, particularly from craft brewing, contain elevated concentrations of hop acids (such as isoα-, α-, and β-acids) [27]. These components exhibit antibacterial, antioxidant, anticancer, and anti-inflammatory properties, rendering spent craft beer yeast a promising ingredient for functional food development [28,29].

### 2.3. Pichia Pastoris

*Pichia pastoris*, a single-celled eukaryotic yeast capable of utilizing methanol as its sole carbon and energy source, is widely used in recombinant protein production and synthetic biology [30]. Yeast hydrolysates, derived from the enzymatic hydrolysis of yeast using proteases, are rich in proteins, minerals, vitamins, and other essential nutrients [31]. These hydrolysates serve as valuable feed additives in livestock and poultry production, supporting microbial cultivation and fermentation.

In animal farming, *Pichia pastoris* is abundant in amino acids, vitamins, and minerals, which promote growth and development, enhance body weight, and improve meat quality. Additionally, it contains a variety of enzymes that facilitate digestion and nutrient absorption, thereby improving feed efficiency and reducing feed waste [31]. Furthermore, it has been shown to improve fecal consistency, reducing emissions of harmful gases such as ammonia and methane, thus contributing to environmentally sustainable livestock farming [30].

### 2.4. Kluyveromyces fragilis

Yeast products derived from *Kluyveromyces fragilis* (*K. fragilis*) have gained increasing attention in industrial production due to their advantageous physiological characteristics and economic potential. These characteristics include rapid growth rate, thermotolerance, the ability to assimilate diverse sugars, and the secretion of hydrolytic enzymes, making them valuable in various applications [32,33].

*K. fragilis* is an active dry yeast, which is rich in proteins, amino acids, vitamins, and minerals. These nutrients play a pivotal role in supporting the growth, immune system, and digestive health of livestock and poultry. The protein and amino acid content in *K. fragilis* ranges from 30% to 40%, providing essential nutrients that promote growth performance and weight gain [34]. Additionally, its vitamins and minerals content contributes to enhanced immune function, improving disease resistance, and reducing the incidence of infections [15]. Furthermore, the active dry yeast of *K. fragilis* facilitates digestion, promotes nutrient absorption, and lowers the risk of digestive disorders, thereby improving overall feed efficiency.

### 2.5. Kluyveromyces marxianus

*Kluyveromyces marxianus* (*K. marxianus*) is one of the fastest-growing yeast species, exhibiting a maximum growth rate of 0.80 h^−1^. It demonstrates exceptional thermotolerance, surviving temperatures up to 52 °C, and can thrive in highly acidic conditions as low as pH 2.3. Furthermore, *K. marxianus* possesses the metabolic versatility to utilize a broad range of carbon sources, including xylitol, glycerol, inulin, and lactose, as well as cost-effective agricultural and industrial by-products such as cheese whey, lemon peel, sugarcane bagasse, and rice bran [35].

Beyond its metabolic adaptability, *K. marxianus* exhibits probiotic properties, particularly through its efficient fermentation of lactose, offering potential benefits for animals with lactose intolerance. It also produces enzymes such as β-galactosidase, which play a crucial role in promoting digestion and nutrient assimilation [36]. In animal husbandry, *K. marxianus* contributes to improved digestion, enhanced immune function, and optimized gut health, thereby reducing reliance on antibiotics. Furthermore, it aids in the degradation of organic waste, enhances nutritional profiles, and mitigates methane emissions, supporting environmentally sustainable animal agriculture. When co-cultured with *S. cerevisiae*, *K. marxianus* enhances lipid metabolism and rumen development by increasing oxidative phosphorylation and ATP production, further promoting sustainable livestock production [37]. These attributes underscore its potential as a valuable feed additive for improving both animal performance and environmental sustainability.

### 2.6. Saccharomyces boulardii

*Saccharomyces boulardii* (*S. boulardii*) is a probiotic yeast widely utilized to enhance the nutritional and functional properties of food and feed. It exhibits low antibiotic resistance, high tolerance to gastrointestinal conditions, notable antioxidant activity, and broad-spectrum antimicrobial properties. *S. boulardii* produces numerous bioactive metabolites, including phenyllactic acid and 2-hydroxyisocaproic acid, which exhibit antioxidant, antimicrobial, antitumor, and anti-inflammatory effects [38].

*S. boulardii* possesses distinct phenotypic and physiological characteristics that contribute to its efficacy as a probiotic, including optimal growth temperature, resistance to gastric conditions, and viability in low pH environments [5]. It is commonly applied in the treatment of inflammatory bowel disease and antibiotic-associated diarrhea. Additionally, *S. boulardii* promotes the proliferation of beneficial gut bacteria, enhances intestinal barrier integrity, and reduces inflammation and oxidative stress in both the gut and brain. Notably, it plays a protective role in maintaining hippocampal cholinergic neuron function and mitigating cognitive decline associated with gut dysbiosis [39].

## 3. Yeast Bioactive Compounds

**Figure 1:**
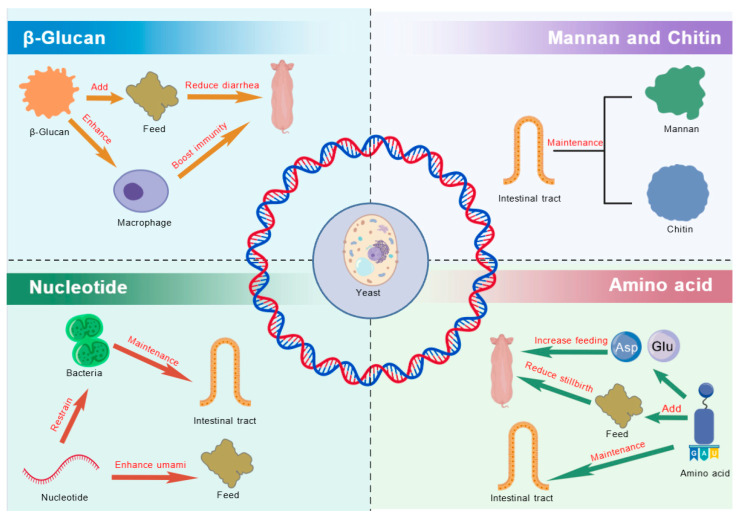
The principal bioactive compounds in yeast. ASP, aspartic acid; Glu, glutamate.

The yeast cell wall comprises approximately 15–20% of the cell’s dry weight [40] and is primarily composed of three major polysaccharides: glucans, mannoproteins, and chitin. Additionally, it contains other active components, including nucleotides, amino acids, and peptides (Figure 1).

Research has demonstrated that yeast polysaccharides exhibit multiple biological functions, including anti-inflammatory effects, immune modulation, regulation of lipid metabolism, and enhancement of gut health [41,42]. The yeast also provides a cell wall, consisting of 55–65% β-glucans, 35–40% mannoproteins, and 2% chitin, which has been shown to enhance immune function, protect skin cells, and exhibit antitumor and antioxidant properties [41,42].

### 3.1. β-Glucan

β-Glucan is a non-starchy polysaccharide widely present in yeast, mushrooms, bacteria, algae, barley, and oats [21]. It consists of β-glycosidic bonds and primarily exists in the forms of (1→3)(1→6)-β-D-glucan, (1→3)(1→4)-β-D-glucan, and (1→3)-β-D-glucan, serving as an essential component of yeast cell walls [43,44].

In pig production, β-glucan exhibits antimicrobial properties and immune-modulating effects. For instance, previous studies have shown that β-glucan derived from *S. cerevisiae* has a thick cell wall (~115 nm) and small particle size, allowing it to interact efficiently with the host immune system [10,11]. This interaction enhances macrophage colonization and activates the antimicrobial activity of monocytes and neutrophils.

Similarly, supplementing weaned piglet diets with 0.1% β-glucan and 0.02% vitamin E improved gut microbiota and reduced the incidence of diarrhea, thereby enhancing growth performance [45]. Additionally, dietary supplementation of β-glucan in piglet feed has been shown to mitigate the negative effects of weaning, improve fecal consistency, and reduce diarrhea incidence in piglets [46].

Beyond gut health, β-glucan promotes the proliferation of beneficial gut microbiota, such as *Roseburia* and *Succinivibrio*, which contribute to increased production of short-chain fatty acids (SCFAs) like butyrate [47]. Furthermore, dietary β-glucan supplementation in weaned piglets significantly improves average daily gain (ADG) and average daily feed intake (ADFI). More importantly, β-glucan modulates gut microbiota composition and metabolic activity, thereby enhancing immune function, supporting growth performance, and alleviating intestinal damage induced by lipopolysaccharide (LPS) challenge [48].

### 3.2. Mannan

Mannan is a key structural component of the yeast cell wall, primarily found in its outer layer. It consists of approximately 90% mannose and 10% protein. The backbone of mannan is composed of mannose residues linked by α-(1→6) glycosidic bonds, with side chains formed by mannose molecules connected via α-(1→2) and α-(1→3) glycosidic bonds [49].

In pig production, dietary supplementation with mannan has been shown to enhance the immune function, reduce inflammation, and improve overall production efficiency. Notably, mannan plays a crucial role in alleviating weaning stress in piglets. For instance, studies have demonstrated that dietary mannan supplementation increases lymphocyte proliferation in the mesenteric and ileal lymph nodes, leading to a stronger immune response and a reduced impact of *Escherichia coli* (*E. coli*) F4 infection [50].

Mannan also contributes to gut health by promoting intestinal development. Research indicates that mannan supplementation increases duodenal villus height and downregulates the expression of inflammation-related genes, such as ileal Peyer’s patches, thereby reducing inflammation and improving the feed conversion ratio (FCR) [12]. Additionally, although dietary mannan supplementation does not always significantly improve the ADG or FCR in weaned piglets, it has been shown to reduce the colonization of cecal pathogens, thereby supporting intestinal health [13].

### 3.3. Chitin

Chitin is a naturally occurring polysaccharide composed of repeating units of N-acetyl-D-glucosamine, a derivative of glucose. Structurally, it closely resembles cellulose but includes an acetyl group attached to the amino group of each glucose unit [51]. As the second most abundant biopolymer in nature, chitin possesses several beneficial properties, including non-toxicity, abundant availability, ease of modification, biodegradability, biocompatibility, and antimicrobial activity. These characteristics make it an attractive material for applications in tissue engineering, drug delivery, and functional feed additives [52].

In pig production, chitin and its derivatives, such as chitosan, have been shown to support growth and enhance gut health in weaned piglets. For instance, dietary supplementation with 500 mg/kg of chitosan has been reported to improve growth rates by increasing serum growth hormone levels and enhancing small intestinal morphology [9]. Additionally, chitin derivatives, particularly chitin oligosaccharides and chitosan oligosaccharides, exhibit anticancer and anti-inflammatory properties, effectively supporting the overall growth and development of piglets [53].

### 3.4. Yeast Nucleotides

Yeast nucleotides are naturally occurring nucleotides found in yeast cells, known for their high bioavailability and rapid absorption in animals. When included in animal feed, yeast nucleotides not only enhance the umami flavor, improving feed palatability and increasing appetite, but also promote digestion and nutrient absorption [14,15,16]. Additionally, yeast nucleotides exhibit immunomodulatory and antioxidant properties. For example, maternal supplementation with yeast nucleotides has been shown to regulate placental nutrient transport via the phosphorylated-mammalian target of rapamycin1-peroxisome proliferator-activated receptor (mTORC1-PPAR) pathway. This influences the metabolism of nucleotides, amino acids, and fatty acids in the liver of newborn piglets, ultimately improving sow reproductive performance [54]. Additionally, yeast nucleotides contribute to intestinal health by mitigating damage caused by *Eimeria* infection and modulating the cecal microbiome [55]. Similar studies indicate that dietary nucleotide supplementation in weaned piglet diets improves gut health by regulating local immune responses and enhancing intestinal mucosal development [6].

### 3.5. Amino Acids

Yeast hydrolysates are rich in amino acids, particularly the eight essential amino acids, with lysine and methionine being the most abundant. Additionally, aspartic acid and glutamic acid contribute to a savory taste, enhancing feed palatability. Dietary supplementation with L-arginine has been shown to increase ADFI, enhance uterine and placental growth, improve nutrient transport, maternal growth, and support maternal health. It also promotes embryo survival, increases piglet birth weight and growth, and improves overall productivity while reducing stillbirth rates [7].

To mitigate the adverse effects of weaning stress, researchers suggest reducing dietary crude protein levels and adjusting amino acid ratios to enhance nutrient digestibility during the fattening phase. For example, replacing dietary antibiotics with 0.20% L-glutamine in nursery pig diets has been found to promote gut microbial diversity [56]. Moreover, reducing dietary protein content by 3% while supplementing it with the appropriate amino acids does not compromise intestinal barrier function in piglets [57]. Instead, it increases the abundance of beneficial bacteria, such as *Lactobacillus* and *Bifidobacterium*, optimizing the gut microbiome and supporting intestinal health.

Furthermore, yeast amino acids exhibit certain antibacterial properties. The supplementation of functional amino acids—primarily threonine, methionine, and tryptophan—has been shown to alleviate the negative effects of plant-based nursery diets on pigs’ immune response to *Salmonella typhimurium* infection [58].

## 4. The Biological Functions of Yeast and Its Derivatives in Animal Husbandry

### 4.1. Appetite-Stimulating Effect

**Figure 2:**
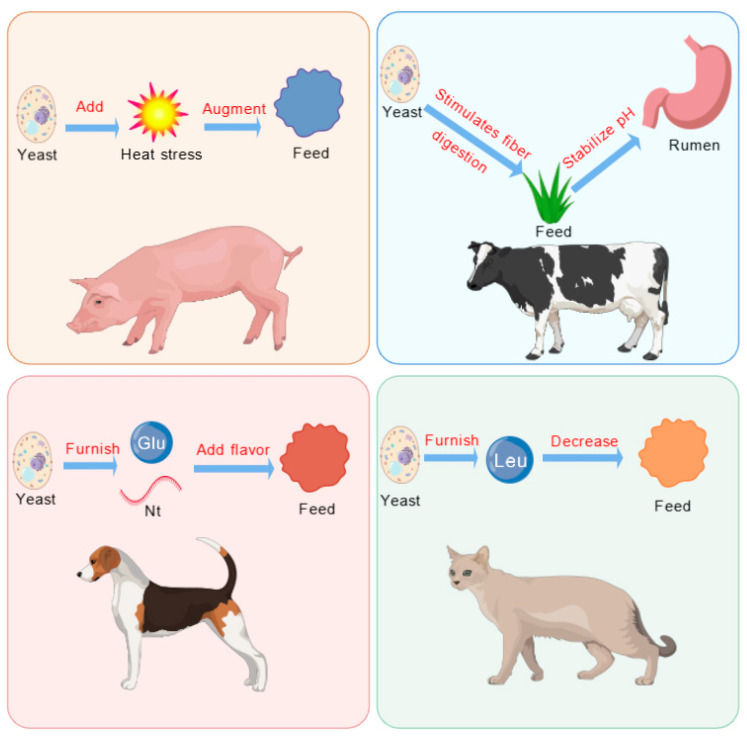
Feeding induction of yeast and its derivatives. Glu, glutamate; Leu, leucine; Nt, nucleotide.

Yeast and its derivatives are processed through techniques such as raw material selection, fermentation, enzymatic hydrolysis, and seasoning. These processes enhance their sensory, nutritional, and functional properties. These characteristics significantly improve the palatability of animal feed, making it more appealing and desirable to livestock and poultry (Figure 2).

The appetite-stimulating effects of yeast and its derivatives operate through multiple mechanisms. Firstly, yeast-derived compounds such as glutamic acid and nucleotides act as flavor enhancers, stimulating taste receptors and increasing feed palatability [59,60]. Secondly, yeast components, particularly β-glucans and mannan oligosaccharides (MOS), promote the growth of beneficial bacteria (*Lactobacillus* and *Bifidobacterium*), improving nutrient absorption and gut health, which indirectly stimulates appetite [61]. Thirdly, yeast hydrolysates contain bioactive peptides that interact with gut–brain axis signaling pathways, influencing satiety and hunger hormones such as ghrelin and leptin [62], thereby regulating appetite and feed intake. Research has demonstrated that incorporating yeast-based products into animal feed improves redox balance and intestinal morphology in calves, while also enhancing innate immunity and stress resistance, helping animals combat infections and dietary challenges [63].

The effects of yeast supplementation on appetite vary among different animal species. In dairy cow diets, yeast hydrolysates do not significantly alter feed intake over time [64]. However, in dog diets, yeast or yeast by-products enriched with glutamic acid and nucleotides enhance feed flavor and increase feed intake [65]. In contrast, in cat diets, the high leucine content in yeast extract may lead to the rejection of “bitter” amino acids, reducing feed intake. Supplementing calf diets with 1.5% and 3% yeast cultures (YC) significantly improves dry matter intake (DMI), ADG, and FCR, promoting calf feeding and growth [66]. Adding yeast mixtures to diets containing large amounts of dried distillers’ grains enhances feed flavor and palatability, thereby increasing ADFI in fattening beef cattle [67]. In dairy cow diets, yeast supplementation increases FCR, while also improving milk yield and fat content, likely due to yeast’s ability to stimulate fiber digestion and stabilize rumen pH [68]. Experimental studies have shown that dietary yeast supplementation does not affect feeding behavior and significantly increases circulating serum transaminase concentrations, indicating that such supplementation has no negative impact on calf performance, carcass characteristics, or feed intake [69].

In summary, yeast and its derivatives serve as exceptional appetite stimulants in livestock and poultry production. Through flavor enhancement, gut microbiota modulation, and gut–brain axis signaling, they improve feed utilization, promote animal growth and development, and represent a high-quality feed additive with broad applications in animal nutrition. Further research is needed to explore the specific molecular pathways involved and optimize yeast derivatives applications across different species and production systems.

### 4.2. Anti-Inflammatory Effects

**Figure 3:**
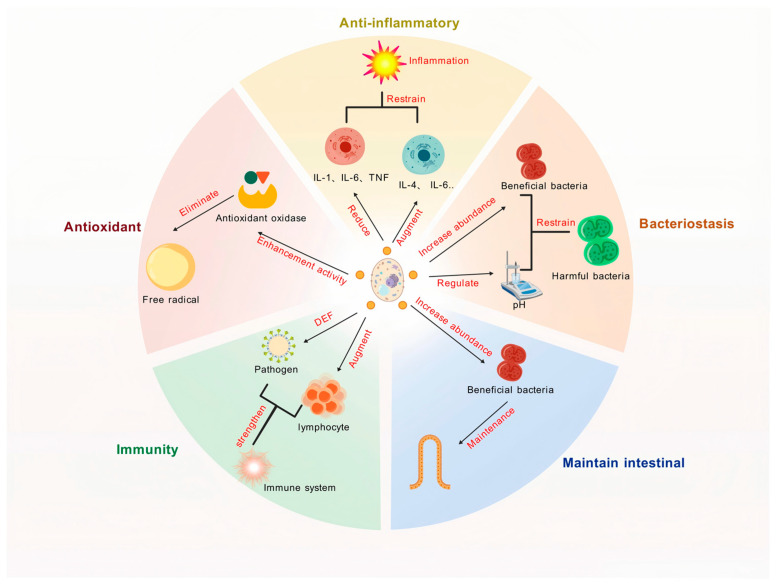
The biological functions of yeast and its derivatives. DEF, defense; IL, interleukin; TNF, tumor necrosis factor.

Yeast and its derivatives have emerged as promising agents for mitigating inflammatory responses, thereby providing a natural strategy to improve animal health. Their anti-inflammatory properties are mediated through multiple mechanisms, including the modulation of cytokine expression, oxidative stress reduction, and gut microbiota regulation, which collectively contribute to alleviating inflammation and improving animal well-being (Figure 3).

One of the primary mechanisms through which yeast and its derivatives exert anti-inflammatory effects is the suppression of pro-inflammatory cytokine expression. Specific yeasts, such as *S. boulardii* and *S. cerevisiae*, have been shown to downregulate key pro-inflammatory cytokines. In patients undergoing colectomy, supplementation with *S. boulardii* significantly reduced mucosal expression of interleukin 1-β (IL-1β) and IL-23A, while also lowering the risk of postoperative infections [70]. Similarly, *S. cerevisiae* has been shown to suppress the upregulation of IL-1β and IFN-γ induced by dextran sulfate sodium, with certain strains restoring the mRNA expression levels of transforming growth factor-β (TGF-β) in the colon to near-normal levels in colitis mouse models [71].

Beyond cytokine modulation, yeast β-glucans play a pivotal role in reducing inflammation by inhibiting oxidative stress, inflammatory mediators, and pro-inflammatory cytokines. These polysaccharides contribute to maintaining the integrity of the intestinal barrier by increasing tight junction proteins (TJP) and modulating the production of SCFAs by the gut microbiota. These combined effects have been shown to alleviate inflammatory conditions in colitis mouse models [72]. Additionally, in rat models, oral administration of β-glucans reduced their levels of inflammatory cytokines, myeloperoxidase, and inducible nitric oxide synthase (iNOS) following lipopolysaccharide (LPS)-induced mastitis. This protective effect extended to the liver and spleen, preventing tissue damage and enhancing the ability to control infections and inflammatory processes [73].

Yeast fermentation products incorporated into animal diets also contribute to reducing inflammation by downregulating the expression of genes involved in microtubule movement, potentially limiting viral transport and subsequent inflammation response [74]. In broiler diets, the inclusion of autolyzed yeast has been shown to enhance antioxidant enzyme activity, increase glutathione (GSH) levels, and reduce the release of inflammatory mediators while inhibiting lipid peroxidation and metal bioaccumulation [75]. Furthermore, supplementation with yeast hydrolysates lowers the expression of TNF-α, IL-1β, and alkaline phosphatase genes in the intestines of broilers, effectively suppressing inflammatory responses and promoting gut health [76].

In summary, yeast and its derivatives exhibit significant anti-inflammatory effects in livestock and poultry production, providing a multifaceted approach to maintaining animal health. By inhibiting pro-inflammatory cytokines expression, reducing oxidative stress, modulating gut microbiota composition, and protecting against tissue damage, yeast-derived compounds provide a natural and effective strategy for mitigating inflammation and enhancing overall animal welfare.

### 4.3. Antioxidant Effects

Yeast and its derivatives have emerged as potent agents in enhancing the antioxidant capacity of livestock and poultry, offering a natural and effective approach to mitigating oxidative stress and improving animal health. Their antioxidant properties are mediated through multiple mechanisms, including the activation of antioxidant enzymes, scavenging of free radicals, and modulation of oxidative stress pathways, which collectively contribute to maintaining redox balance and promoting animal well-being (Figure 3).

One of the primary mechanisms through which yeast and its derivatives exert antioxidant effects is their ability to directly neutralize reactive oxygen species (ROS). Yeast peptides have been shown to exhibit strong antioxidant activity, with experimental studies demonstrating that waste beer yeast peptides at a concentration of 35 mg/mL can effectively scavenge hydroxyl radicals, 1,1-diphenyl-2-picryl-hydrazyl radical, and 2,2′-azino-bis(3-ethylbenzothiazoline-6-sulfonic acid) with efficiencies of 95.10%, 98.37%, and 69.41%, respectively, highlighting their potent free radical-neutralizing capabilities [77].

Additionally, yeast derivatives, particularly selenium-enriched yeast (SY), significantly boost the activity of key antioxidant enzymes such as glutathione peroxidase (GSH-Px), catalase (CAT), and superoxide dismutase (SOD). For instance, SY protein hydrolysates have been shown to alleviate skin damage and oxidative stress induced by ultraviolet B radiation by increasing GSH-Px and CAT activity, as well as GSH levels in the skin or serum [78]. Dietary supplementation of 1.5 mg/kg SY in laying hens significantly enhances egg quality, promotes selenium deposition, and improves antioxidant capacity, while optimizing liver gene expression. At this dosage, SY maintains normal protein metabolism and immune function without causing adverse effects on liver health. Studies have demonstrated that SY supplementation markedly upregulates the expression of key antioxidant-related genes (Nrf2, HO-1, and NQO1) and downregulates Keap1 expression. Moreover, SY elevates the serum antioxidant enzymes activity (GSH-Px, SOD) and total antioxidant capacity (T-AOC), thereby strengthening the defense mechanisms against oxidative stress [79].

Yeast supplementation has also been shown to reduce oxidative stress by lowering malondialdehyde (MDA) levels, a marker of lipid peroxidation. In layer diets, yeast culture (YC) significantly increases serum T-AOC and GSH-Px activity while reducing MDA levels, thereby improving antioxidant capacity and egg quality [80]. Similarly, in broiler diets, the addition of SY enhances GSH levels and trends toward increasing T-AOC, SOD, and CAT activity while decreasing MDA levels [81]. This selenium-mediated enhancement of antioxidant enzyme activity provides robust protection against oxidative damage. YC, enriched with vitamins, mannans, oligosaccharides, and selenium, contributes to improving antioxidant capacity. For example, in Sichuan white geese diets, dietary YC supplementation enhances serum GSH-Px activity, thereby strengthening antioxidant defenses [82].

Beyond its role in antioxidant defense, yeast supplementation has been linked to improved metabolic health and performance in dairy animals. A study has demonstrated that supplementing YC in the diet of lactating dairy goats modulates the rumen microbial community, leading to enhanced total protein, glucose, creatinine, as well as SOD and catalase CAT activity. These metabolic and enzymatic improvements contribute to better rumen fermentation, enhanced serum metabolism and antioxidant capacity, and ultimately improved lactation performance [83]. Furthermore, *K. marxianus*, enriched with β-glucans, has been shown to coordinate with sulfasalazine in reducing colonic inflammation. This therapeutic effect is mediated by the beneficial regulation of inflammatory cytokines, inflammatory mediators, and tight junction proteins (TJP), along with the restoration of gut microbial balance [84].

In summary, yeast and its derivatives exhibit significant antioxidant effects in livestock and poultry production, providing a multifaceted approach to mitigating oxidative stress and improving animal health. By enhancing antioxidant enzyme activity, scavenging free radicals, and reducing oxidative stress markers, yeast-derived compounds represent a natural and effective strategy for maintaining redox balance and promoting overall animal well-being.

### 4.4. Antimicrobial Effects

Yeast and its derivatives are not only pivotal in enhancing the antioxidant capacity of animals but also exhibit robust antimicrobial properties, making them essential tools in reducing pathogen infections and transmission during livestock and poultry production. These antimicrobial effects are mediated through diverse mechanisms, including modulation of gut microbiota, enhancement of intestinal barrier function, and immune system regulation, which collectively contribute to improved animal health and productivity (Figure 3).

Yeast components, such as β-glucans and mannan oligosaccharides (MOS), play a critical role in reshaping the gut microbial community. For instance, β-glucans increase the number of goblet cells, upregulate the expression of TJP in intestinal epithelial cells, and modulate cytokine production, thereby suppressing the growth of intestinal pathogens and enhancing gut health [85]. Similarly, MOS supplementation enhances gut microbiota diversity, increases the population of probiotics, and inhibits the downregulation of intestinal mucosal protein expression and goblet cell reduction following pathogenic *E. coli* infection, significantly protecting organs such as the intestines, liver, spleen, and lungs [86].

Yeast derivatives fortify the intestinal barrier, an essential defense against pathogen invasion. By stimulating goblet cell proliferation and promoting TJP expression, yeast components help maintain mucosal integrity, reducing the likelihood of infection [85]. This mechanism is particularly effective in mitigating the adverse effects of pathogenic infections.

Yeast components also stimulate the immune system by modulating cytokine production and enhancing the expression of immune-related genes. For example, supplementing β-glucans in poultry diets significantly increases the mRNA expression of IL-10, IL-17F, IFN-γ, and inducible iNOS in the thymus, thereby providing protective effects against coccidiosis infection in broilers [87].

Yeast fermentation products also play a role in suppressing pathogenic bacteria by altering gut pH and producing antimicrobial metabolites such as SCFAs. In broiler diets, fermented feed containing yeast is shown to increase cecal concentrations of SCFAs—including acetate, propionate, butyrate, and lactate—while lowering cecal pH, creating an unfavorable environment for pathogenic bacteria and promoting the proliferation of beneficial bacteria [88]. In ruminants, yeast culture (YC) supplementation modifies the rumen environment by reducing ammonia nitrogen concentration and SCFA levels, which suppresses the growth of harmful bacteria while favoring beneficial bacteria populations [89].

In general, yeast and its derivatives exert significant antimicrobial effects in livestock and poultry production through multiple mechanisms, including gut microbiota modulation, intestinal barrier enhancement, immune system regulation, and production of antimicrobial metabolites. These properties not only reduce the risk of pathogen infections and disease transmission but also improve animal health, growth performance, and production efficiency. By leveraging the antimicrobial potential of yeast, the farming industry can enhance sustainable livestock production while minimizing the reliance on antibiotics.

### 4.5. Maintaining Intestinal Health

Yeast and its derivatives play a critical role in maintaining intestinal health in livestock and poultry production. As the largest immune organ in animals, the gut directly impacts growth performance, immune function, and overall well-being (Figure 3). Through microbiota modulation, enhancement of gut barrier integrity, and immune regulation, yeast-based supplements contribute to improved digestion, nutrient absorption, and disease resistance.

One of the key ways yeast supports intestinal health is by promoting the growth of beneficial gut bacteria while suppressing pathogenic microbes. For example, studies have shown that supplementing breeder hen diets with 2.0 g/kg of yeast culture (YC) improves the structure of the gut microbiota by increasing *Lactobacillus* abundance and bacterial community stability, thereby enhancing feed digestion, absorption, and intestinal health [90]. Similarly, the addition of brewer’s yeast autolysate to the diet of gilthead seabream significantly enhances microbiota regulation by increasing beneficial bacteria capable of degrading indigestible carbohydrates and producing SCFAs [91]. In Texas donkey diets, the inclusion of 0.1% yeast polysaccharides promotes beneficial gut bacteria proliferation and increases microbial diversity, further supporting intestinal health [92].

Yeast derivatives also contribute to gut health by improving intestinal morphology and function. In carp, the dietary inclusion of 3% solid-state fermentation products of yeast (SFPY) improves both intestinal and hepatic health by reducing lymphocyte infiltration and enhancing villus growth. Furthermore, SFPY modulates gut microbiota composition by increasing *Cetobacterium* abundance and reducing *Shewanella* populations [93]. Similarly, the intestinal health of salmon fed with yeast cell wall extract (YCWE) is improved by regulating inflammation-related genes, enhancing antioxidant capacity, and upregulating the expression of intestinal tight junction proteins. Additionally, YCWE positively modulates the intestinal microbiota of sea bass by increasing beneficial bacteria, such as *Cetobacterium* [94].

Yeast supplementation further enhances fiber digestion and fermentation efficiency in herbivores. In rabbits, active yeast supplementation leads to a gradual increase in the relative abundance of Firmicutes in the cecal microbiota, which enhances the digestion of high-fiber feeds and promotes antimicrobial substances production in the gut [95]. In fattening Hu sheep, dietary supplementation with 20 and 40 g/d of YC improves nutrient digestion, particularly nitrogen utilization, while enhancing the rumen microbial environment, and epithelial development, thereby enhancing growth performance and slaughter yield [96].

In broilers, yeast derivatives promote gut structure development and immune defense. Yeast hydrolysates improve jejunal villus height and the villus height-to-crypt depth (V/C) ratio while reducing crypt depth, with a 100 mg/kg supplementation level yielding better results than 150 mg/kg [76]. Yeast β-glucans also reduce diarrhea incidence in young rabbits, strengthen intestinal mucosal defenses, and stabilize gut microbiota [97]. Additionally, dietary yeast peptides reduce the relative abundance of unfavorable bacteria in the broiler ileum while increasing beneficial microbial populations, leading to a healthier gut environment [98].

Yeast-based interventions also restore gut microbial balance and enhance intestinal metabolism. Supplementation with *K. marxianus* has been shown to inhibit *Candida* overgrowth while enhancing *Epicoccum* abundance, thereby preventing dysbiosis and promoting gut health [99]. Another study demonstrated that *K. marxianus* hydrolysate supplementation boosts microbial diversity and evenness, elevates *Lactobacillus* and *Akkermansia* levels, and modulates cecal metabolic pathways, especially those involved in energy and amino acid metabolism, contributing to significant improvements in gut health [100].

In summary, yeast and its derivatives play an essential role in maintaining intestinal health in livestock and poultry production. Through gut microbiota modulation, gut barrier enhancement, and immune regulation, yeast-based supplements contribute to improved digestion, nutrient absorption, and disease resistance.

### 4.6. Immune Regulation

Yeast and its derivatives play multifaceted and crucial roles in enhancing the immune response and overall health of livestock and poultry through various mechanisms (Figure 3). These mechanisms include the activation of innate and adaptive immunity, regulation of cytokine production, and enhancement of gut-associated immune responses, all of which contribute to improved disease resistance and overall health.

Firstly, β-glucans, as a key component, have been shown to activate the secretion of β-defensin, IL-6, and IL-10 in sheep rumen explants, thereby promoting mucosal immunity and enhancing the local immune defense against pathogens [101]. Furthermore, β-D-glucans in hydrolyzed yeast bind to specific carbohydrate structures on epithelial surfaces and macrophage receptors, triggering a cascade reaction that activates macrophages and stimulates the release of cytokines, which in turn strengthens the acquired immune responses [102].

Cellular adaptive immunity is also significantly influenced by yeast supplementation. Changes in lymphocyte subsets, such as helper T lymphocytes (CD4^+^ CD8^−^) and cytotoxic T lymphocytes (CD4^−^ CD8^+^), serve as important markers for immune system activation. An increase in these lymphocyte populations indicates a heightened immune state, contributing to improved disease resistance [103].

The application of yeast hydrolysates in the diet of Nile tilapia fry increases the height of midgut epithelial villi and the number of intraepithelial lymphocytes, improving both nutrient absorption and microbial antigen clearance [104]. This indicates a direct link between yeast hydrolysates, gut health, and immune function.

Supplementing calf diets with yeast hydrolysates significantly increases serum levels of TNF-α and IL-1β, while elevating concentrations of acute-phase proteins such as haptoglobin, serum amyloid A, and transferrin. These immune markers indicate a strengthened innate immune response and a reduced stress reaction in calves, leading to better health and resilience against infections [105]. Similarly, in broiler breeder chicks, yeast hydrolysates improve liver function by reducing aspartate aminotransferase concentrations in both the spleen and plasma. This reduces the risk of liver diseases, muscle damage, cardiovascular issues, and kidney injuries, while moderating immune and metabolic functions [106]. In goat diets, the addition of yeast improves the immune system by increasing white blood cell counts, supporting a stronger immune defense and overall health maintenance [107].

In general, yeast and its derivatives exert significant immunomodulatory effects in livestock and poultry, effectively improving animal health and production performance.

## 5. Application of Yeast and Its Derivatives in Pig Production

### 5.1. Sows

The application of yeast and its derivatives in sow production has demonstrated significant benefits, improving reproductive performance, gut health, immune function, and overall well-being (Table 1). These benefits are achieved through various mechanisms, including enhanced nutrient digestibility, modulation of gut microbiota, immune regulation, and oxidative stress reduction.

#### 5.1.1. Enhancing Reproductive Performance and Gut Health

The addition of yeast culture (YC) effectively enhances sow growth performance and overall health. Firstly, supplementing sow diets with 24 g/L and 40 g/L of YC during late gestation and lactation positively influences metabolic hormone secretion, increasing plasma concentrations of glucagon-like peptide-1 and neuropeptide Y, which support gut health and overall physiological status [108]. Additionally, the inclusion of yeast cultures in the diets of primiparous sows and late-gestation sows yields distinct benefits. For example, adding YC to the diets of primiparous sows increases the apparent total tract digestibility of energy and calcium during lactation, enhances acetate and propionate levels during gestation, promotes the growth of beneficial bacteria, and inhibits harmful bacteria, thereby improving reproductive performance and gut health [109].

Similarly, supplementing 0.8% yeast culture in primiparous sows’ diets elevates plasma levels of L-leucine, creatine, and D-proline, which help inhibit partial lipolysis and further enhance reproductive performance [110]. A study noted that maternal supplementation with 4 g/kg of YC during late gestation improves the development of small intestinal villi in newborn piglets, increases the expression of pro-inflammatory and anti-inflammatory cytokines, and enhances secretory Immunoglobulin A (IgA) gene expression, preventing diarrhea and increasing weaning weight [14].

#### 5.1.2. Improving Placental Function and Immune Transfer

Yeast nucleotides have been shown to support placental function and fetal development. Adding 0.4% yeast nucleotides improves placental transport by regulating the mTORC1-PPAR pathway, directly influencing nucleotide, amino acid, and fatty acid metabolism in newborn piglets, reducing birth mortality and intrauterine growth retardation, and thereby enhancing sow reproductive performance [54]. Similarly, dietary supplementation with 200 g/t of nucleotides improves immunoglobulin levels in sow colostrum. Supplementing yeast nucleotides or purified pyrimidines increases feed intake during lactation, while purified purines significantly reduce serum C-reactive protein levels in sows, alleviating systemic inflammation [111].

Yeast postbiotics are gaining recognition for their role in improving feed utilization and gut health. Adding 0.27 to 0.32 g/kg of yeast postbiotics to sow diets can increase ADG and ADFI, while shortening the number of days to estrus [112]. Moreover, supplementing 2% yeast-derived postbiotics during late gestation and lactation increases the Chao1 index and alpha diversity of gut microbiota, promoting beneficial bacteria and stabilizing SCFA production, which is crucial for gut homeostasis [113].

Yeast-derived selenium and postbiotics further contribute to antioxidant defense and immune regulation. Studies indicate that supplementing sow diets with SY increases antioxidant-related microbiota and SCFA-producing microbiota, alters fecal microbial composition, and improves piglet growth performance, selenium status, antioxidant capacity, and immunoglobulin transfer. This ultimately strengthens small intestinal barrier function and activates the Nrf2/Keap1 pathway in offspring [114]. Additionally, supplementing 0.125% yeast-derived postbiotics improves the production performance of sows and their piglets, increases average weaning weight, raises IgG and IgA concentrations in sow milk, and boosts IgG and IgM levels in piglet serum, while reducing piglet mortality and diarrhea incidence [18].

#### 5.1.3. Reducing Oxidative Stress and Enhancing Maternal Health

Yeast extracts and β-glucans contribute to mitigating oxidative stress in sows. Feeding 10 g/kg of yeast extract increases ADFI, reduces weight loss during lactation, improves piglet production performance, and alleviates oxidative stress in both sows and piglets [115]. The addition of 0.01% yeast β-glucan to sow diets can reduce the occurrence of oxidative stress by lowering the respiratory rate and hair cortisol levels, as well as decreasing the levels of TNF-α, LPS, and SOD [116]. Similarly, dietary supplementation with selenium yeast and glycerol monolaurate during gestation helps improve placental development and function, thereby alleviating intrauterine oxidative stress and inflammation [117]. Live yeast supplementation in maternal diets has been linked to improved gut microbiota composition and reduced stillbirths. A study showed that maternal supplementation with 1 g/kg of live yeast positively regulates gut microbial communities, improves sow health, and reduces the number of stillborn and low-birth-weight piglets [3]. Similarly, adding 2% yeast single-cell protein in sow diets has been shown to significantly enhance reproductive performance, reduce the population of harmful pathogenic microorganisms in the intestinal tract, and consequently improve the growth performance of piglets [118].

### 5.2. Weaned Piglet

The application of yeast and its derivatives in piglet production has shown significant effects in improving growth efficiency and antioxidant capacity. These natural additives function through multiple mechanisms, including microbiota modulation, immune enhancement, and oxidative stress reduction, ultimately supporting overall piglet well-being (Table 2).

#### 5.2.1. Enhancing Gut Health and Microbial Balance

The cell walls of *S. cerevisiae*, particularly mannans and β-glucans, influence microbial populations in the gastrointestinal tract. Mannans can bind to bacterial adhesins, preventing pathogenic bacteria from colonizing the gut and thereby altering the gut microbial environment and reducing the incidence of diarrhea [119]. Studies have also shown that adding molasses yeast powder rich in 7.5% β-glucans to the diet enhances piglets’ antibody response against swine fever virus, by modulating Peyer’s patch cells in the intestinal epithelium or increasing the number and function of intraepithelial lymphocytes [120].

Maternal yeast supplementation also benefits offspring. For example, supplementing the sow with 0.2% yeast culture downregulates IL-6 and IL-10 expression in the thymus of weaned piglets and reduces the activity of c-Jun N-terminal kinase in the liver, thereby alleviating inflammatory responses and enhancing immune function [121]. The inclusion of 1% live yeast in sow diets enriches beneficial microbial populations in piglet intestines, leading to improved post-weaning ADG and ADFI [122]. Additionally, feeding gestating and lactating sows with 0.05% to 0.1% live yeast (LY) can enhance offspring intestinal development and health by upregulating TJP and antioxidant enzymes expression [123].

#### 5.2.2. Improving Nutrient Utilization and Digestive Function

Yeast culture (YC) serves as an alternative protein source while enhancing digestive function. Directly adding 10% YC to piglet diets improves feed palatability, increases mucosal digestive enzyme activity and absorption capacity, and strengthens the acidic environment of the gut, thereby inhibiting pathogen growth and maintaining intestinal health [124]. Additionally, YC supplementation in weaned piglets reduces serum diamine oxidase activity, increases jejunal villus height and villus-to-crypt (V/C) ratio, and enhances the mRNA expression and protein abundance of intestinal TJ proteins, lowers pro-inflammatory factor levels in serum and intestinal tissues, reduces oxidative damage, and boosts immunity [109].

Similarly, adding yeast hydrolysates to the diet enhances piglet growth performance and gut integrity. For instance, supplementing diets with 1.0% autolyzed yeast reduces inflammation and stress markers, improves intestinal morphology and structure, and alters gut microbiota by reducing pro-inflammatory bacteria associated with inflammation and obesity [4]. Higher supplementation levels (5%) have been shown to further increase ADG and ADFI of piglets [126]. Additionally, supplementing 1.5% YC in low-protein diets can effectively enhance FCR, boost antioxidant capacity, and promote intestinal microbiota balance [125].

#### 5.2.3. Boosting Antioxidant Capacity and Reducing Oxidative Stress

Yeast derivatives contribute significantly to mitigating oxidative stress in weaned piglets. The addition of 10 g/kg *Kluyveromyces fragilis* hydrolysate increases plasma SOD activity and reduces MDA concentration, enhancing antioxidant capacity [15]. Moreover, the inclusion of a mycotoxin detoxifier composed of curcumin, silymarin, and a yeast-based component has been shown to significantly reduce oxidative stress biomarkers while increasing total antioxidant capacity, thereby enhancing antioxidant activity and improving production performance [134].

Further studies have demonstrated that dietary supplementation with 10 g/kg of autolyzed SC yeast or 1.5 g/kg of sodium butyrate promotes growth performance in weaned piglets by enhancing intestinal barrier integrity, upregulating the mRNA expression of nutrient transporters, and boosting antioxidant enzyme activity [127]. Similarly, supplementing piglet diets with 500 mg/kg yeast peptides optimizes the intestinal environment, increases serum GSH peroxidase levels, and maintains gut health [128].

#### 5.2.4. Strengthening Immunity and Disease Resistance

Yeast polysaccharides and postbiotics have been shown to enhance piglet immunity. Adding 0.3% and 0.45% yeast polysaccharides increases SCFA production (acetate, propionate, and butyrate) in the colon, reduces the abundance of *E. coli* and *Salmonella* in the cecum, and improves intestinal morphology and microbial structure [129]. In addition, the inclusion of 175 g/ton of yeast postbiotics in feed can mitigate the negative effects associated with F18^+^ *E. coli* infection by reducing Gram-negative bacteria abundance, thereby supporting intestinal health and enhancing immune function [132].

Maternal yeast nucleotide supplementation also benefits piglet growth. Studies have shown that adding 1 g/kg yeast nucleotides to sow diets promotes piglet growth, likely due to higher nucleotide concentrations in sow milk, which reduces oxidative stress in both sows and piglets [130]. Direct application of yeast products also significantly impacts piglet growth. Jiang et al. have demonstrated that dietary supplementation with 3.0 g/kg live yeast and 3.0 g/kg ultrafine yeast powder boosts immunity by improving small intestinal morphology (increased duodenal and jejunal villus height and the V/C ratio), and enhances serum IgA concentrations [131].

### 5.3. Growing-Finishing Pigs

Yeast and its derivatives offer multiple advantages in finishing pig production, enhancing production performance, meat quality, immune function, and gut health. These benefits are achieved through improved metabolism regulation, enhanced antioxidant capacity, and microbiota modulation (Table 3).

#### 5.3.1. Enhancing Meat Quality and Growth Performance

The addition of YC has been shown to positively impact meat quality. For example, supplementing growing pig diets with 2% YC reduces body fat and positively impacts intestinal barrier function by improving the richness of beneficial gut bacteria [135].

Similarly, adding 0.5% YC to wheat–rice diets improves nitrogen utilization, boosts antioxidant capacity, enhances the immune function, and increases the marbling score and meat color of finishing pigs. The inclusion of novel *S. cerevisiae* strains has also been reported to improve production performance, immune function, and muscle quality by regulating lipid, carbohydrate metabolism, and starch metabolism [136].

#### 5.3.2. Supporting Heat Stress Adaptation and Energy Metabolism

Live yeast supplementation plays a crucial role in improving the thermotolerance of growing pigs. Maintaining a balance between metabolic heat production and dissipation enhances energy metabolism and thermoregulatory responses. Research has shown that live yeast supplementation increases feeding frequency, improving energy intake and insulin sensitivity, while enhancing heat loss efficiency during heat stress conditions [137].

Additionally, live yeast supplementation regulates the microbiota, influencing feeding behavior and metabolism in pigs, particularly under thermoneutral conditions [138]. Supplementing diets with 0.25% live yeast improves feed efficiency and reduces plasma TNF-α, a pro-inflammatory mediator, thereby decreasing inflammation during heat stress [17].

#### 5.3.3. Modulating Gut Microbiota and Immune Function

Yeast derivatives contribute significantly to gut health and immune regulation. Yeast cell walls (YCW), rich in mannan-oligosaccharides, function as antimicrobial feed additives by binding to pathogens and preventing their colonization, thereby improving gut integrity and immune responses [143]. Supplementing diets with 0.2% yeast cell wall extract (YCWE) enhances intestinal health by increasing jejunal villus height and reducing the proportion of immunoglobulin A and jejunal pathogens [139].

Additionally, hydrolyzed yeast cell wall products have been shown to improve gut barrier integrity in nursery pigs. Supplementing with 0.05% sodium butyrate and 0.1% enzymatically hydrolyzed yeast cell wall products can enhance feed efficiency, reduce jejunal permeability to fluorescein isothiocyanate-dextran 4 kDa, and improve intestinal integrity. Furthermore, dietary hydrolyzed yeast cell wall product increases cecal weight, enhances cecal fermentation capacity, and modulates the microbial composition in both the cecum and feces [143].

#### 5.3.4. Improving Antioxidant Capacity and Lipid Metabolism

Yeast-based supplements also contribute to antioxidant defense and lipid metabolism regulation. Supplementing with 20 mg/kg YC alleviates intestinal villus morphological damage caused by the porcine epidemic diarrhea virus and enhances the antioxidant capacity of serum and small intestines in infected piglets [142]. Additionally, supplementing diets with 200 μg/kg chromium yeast improves meat marbling and juiciness, while also increasing serum IgG and IgM levels, reducing high-density lipoprotein cholesterol, and increasing low-density lipoprotein cholesterol, thereby boosting antioxidant capacity and immune function [16].

Yeast supplementation has also been linked to improved feed efficiency and meat quality. Adding 0.5% autolyzed yeast to pig diets reduces feed intake, improving FCR, blood parameters, and supporting optimal body condition and meat quality in finishing pigs [141].

## 6. Conclusions and Future Prospects

Yeast and its derivatives have emerged as versatile and sustainable solutions in pig production, offering a broad spectrum of biological functions that address key challenges in animal health, growth performance, and productivity. Their proven ability to enhance feed efficiency, improve gut health, regulate immune responses, and mitigate oxidative stress underscores their potential as invaluable alternatives to traditional antibiotics and synthetic growth promoters. Extensive research has validated the efficacy of yeast-based feed additives in reducing pathogen loads and enhancing overall animal health. As the swine industry shifts toward sustainable and efficient production, yeast-derived products are poised to play a pivotal role in enhancing animal nutrition and health management.

Moving forward, further research is essential to optimize the selection and engineering of yeast strains with enhanced bioactive efficacy, explore cost-effective cultivation methods (e.g., utilizing agricultural by-products as carbon sources such as straw), and refine the application strategies of yeast-based products at different production stages. Additionally, a deeper mechanistic understanding of yeast’s functional properties will aid in the development of targeted formulations tailored to the specific physiological and metabolic needs in swine production. By fully harnessing the potential of yeast and its derivatives, the swine industry can achieve greater efficiency, sustainability, and resilience, contributing to improved animal welfare, global food security, and environmental sustainability.

## Figures and Tables

**Table 1:** Dietary applications of yeast and yeast extracts in sow production.

Source	Type	Dosage	Main Results	References
*Saccharomyces cerevisiae*	Selenium yeast	1 g/kg	Beneficially regulated the intestinal microbiota; Improved the health status of sows and reduced the number of stillborn piglets and piglets with low birth weight	[3]
Angel yeast	Yeast nucleotides	4 g/kg	Improved small intestinal development of newborn piglets; Enhanced immunity of newborn piglets and prevented diarrhea	[14]
*-*	Yeast nucleotides	4 g/kg	Regulated placental transport function; Improving reproductive performance	[54]
*Saccharomyces cerevisiae*	Yeast culture	Gestation period: 24 g/L Lactation period: 40 g/L	Increased concentrations of glucagon-like peptide-1 and neuropeptide Y in sow plasma; Improved intestinal health	[108]
*Saccharomyces cerevisiae*	Yeast culture	Gestation period: 5 g/kg Lactation period: 8 g/kg	Improved sow reproductive performance; Increased ATTD of total energy and calcium; Increased fecal SCFA production; Regulated gut microbiota	[109]
*Saccharomyces cerevisiae*	Yeast culture	8 g/kg	Improved reproductive performance; Increased plasma L-leucine, creatine, and D-proline levels	[110]
*-*	Yeast nucleotides	0.2g/kg	Improved immunoglobulin levels,	[111]
*Saccharomyces cerevisiae*	*Saccharomyces* yeast postbiotics	0.27–0.32 g/kg	Increased ADG and ADFI; Improved reproductive performance	[112]
*Saccharomyces* yeast	*Saccharomyces* yeast postbiotics	20 g/kg	Maintained gut microbiota homeostasis; Regulated SCFA production; Promoted intestinal health	[113]
Selenium-enriched yeast	Selenium yeast	0.0002 g/kg	Enhanced fecal SCFA production; Improved growth performance, selenium status, antioxidant capacity, and immunoglobulin transfer of piglets	[114]
*-*	Yeast nucleotides	10 g/kg	Increased ADFI; Reduced weight loss during lactation; Improved piglet production performance; Alleviated oxidative stress in sows and piglets	[115]
*Saccharomyces cerevisiae*	β-glucan	0.1 g/kg	Reduced oxidative stress; Decreased TNF-α, LPS, and SOD levels	[116]
Selenium yeast and glycerol monolaurate supplementation	-	0.0002 g/kg SeY + 1 g/kg GML	Improve placental development and function; Alleviated intrauterine oxidative stress and inflammation	[117]
*Saccharomyces cerevisiae*	Yeast single-cell protein	20 g/kg	Enhanced reproductive performance; Reduced intestinal harmful pathogenic microorganisms; Improved growth performance of piglets	[118]

Notes: ATTD, apparent total tract digestibility; SCFA, short-chain fatty acids; ADG, average daily weight gain; TNF-α, tumor necrosis factor-α; LPS, lipopolysaccharide; SOD, superoxide dismutase.

**Table 2:** Dietary applications of yeast and yeast extracts in weaned piglet production.

Source	Type	Dosage (g/kg)	Main Results	References
*Saccharomyces cerevisiae*	Autolyzed yeast	10	Regulated intestinal microbiota; Suppressed inflammation; Improved intestinal environment	[4]
*Kluyveromyces fragilis*	Yeast hydrolysate	10	Enhanced antioxidant capacity; Reduced oxidative damage	[15]
*Saccharomyces cerevisiae*	Yeast culture	5	Increased serum GSH-Px level; Enhanced antioxidant capacity; Improved gut microbiota health	[109]
*Saccharomyces cerevisiae*	Yeast cell wall	2	Improved intestinal microbiota; Reduced diarrhea rate	[119]
*Saccharomyces cerevisiae*	Yeast powder	75	Enhanced immunity and the number and function of intraepithelial lymphocytes in the gut epithelium	[120]
*-*	Yeast culture	2	Downregulated gene expression of IL-6 and IL-10 in the thymus; Reduced inflammation	[121]
*Saccharomyces cerevisiae*	Live yeast	10	Improved the post-weaning ADG and ADFI of offspring	[122]
*Saccharomyces cerevisiae*	Live yeast	Gestation period: 0.5 Lactation period: 10	Enhanced offspring intestinal development and health; Activated immune system; Upregulated TJP and antioxidant enzymes gene expression	[123]
*Saccharomyces cerevisiae*	Yeast culture	100	Inhibited pathogens’ growth; Improved intestinal health	[124]
*Saccharomyces cerevisiae*	Yeast culture	15	Enhanced FCR and antioxidant capacity; Promoted intestinal microbiota balance in piglets fed low-protein diets	[125]
*Saccharomyces cerevisiae*	Autolyzed yeast	50	Enhanced ADG and ADFI of piglets; Improved their growth performance	[126]
*Saccharomyces cerevisiae*	Autolyzed yeast; Sodium butyrate	10 autolyzed yeasts or 1.5 sodium butyrate	Improved growth performance in weaned piglets; Enhanced intestinal barrier integrity; Boosted antioxidant enzyme activity	[127]
*Saccharomyces cerevisiae*	Yeast peptides	0.5	Increased serum GSH-Px level; Enhanced antioxidant capacity; Improved gut microbiota health	[128]
*Saccharomyces cerevisiae*	Yeast polysaccharides	3; 4.5	Improved production performance; Increased colonic SCFAs levels; Reduced E. coli and Salmonella in the cecum; Enhanced intestinal development	[129]
*-*	Yeast nucleotides	1	Decreased oxidative stress	[130]
*Saccharomyces cerevisiae*	Live yeast; Yeast powder	3; 3	Enhanced intestinal development; Increased serum IgA level and enhanced immunity	[131]
*-*	Yeast postbiotics	0.175	Mitigated the negative effects of E. coli infection; Enhanced intestinal immune function	[132]
*Saccharomyces*	Yeast postbiotics	0.175	Alternative protein sources provide numerous nutritional benefits	[133]
*-*	Curcumin/Silymarin/Yeast-based	25	Reduced oxidative stress biomarkers; Enhanced antioxidant activity; Improved production performance	[134]

Notes: IL-6, interleukin-6; IL-10, interleukin-10; FCR, feed conversion ratio; GSH-Px, glutathione peroxidase; SCFAs, short-chain fatty acids; IgA, immunoglobulin A; ADG, average daily gain; ADFI, average daily feed intake; TJP, tight junction proteins.

**Table 3:** Application of yeast and yeast extracts in finishing pig production.

Source	Type	Administration Method	Dosage	Main Results	References
Chromium yeast	Chromium yeast	Added to feed	200 μg/kg	Increased serum IgG and IgM levels; Improved antioxidant capacity and immune ability; Reduced backfat thickness	[16]
*Saccharomyces cerevisiae*	Live yeast	Added to feed	2.5 g/kg	Improved feed efficiency; Reduced inflammation	[17]
*Saccharomyces cerevisiae*	Yeast culture	Added to feed	20 g/kg	Reduced body fat; Enhanced intestinal barrier function	[135]
*Saccharomyces cerevisiae*	Yeast culture	Added to feed	1 × 10^6^ CFU/g	Improved nitrogen utilization and antioxidant capacity; Regulated immunity and microbial community; Improved meat color	[136]
*Saccharomyces cerevisiae*	Live yeast	Added to feed	1 × 10^6^ CFU/g	Improved insulin sensitivity and heat loss efficiency, increased feed intake	[137]
*Saccharomyces boulardii*	Live yeast	-	1 × 10^6^ CFU/g	Improved heat stress; Increasing daily meal frequency	[138]
*Saccharomyces cerevisiae*	Yeast cell wall	Added to feed	2 g/kg	Increased jejunum villus height; Reduced the proportion of IgA	[139]
*Saccharomyces cerevisiae*	Yeast cell wall; Sodium butyrate	Added to feed	0.5 g/kg sodium butyrate, 1 g/kg Yeast cell wall	Enhanced FCR; Reduced jejunal permeability to FITC-Dextran 4 kDa; Improved intestinal integrity; Modulated the microbial composition in both the cecum and feces	[140]
*Saccharomyces cerevisiae*	Autolyzed yeast	Added to feed	5 g/kg	Reduced ADFI; Improved FCR and meat quality	[141]
-	Yeast culture	Oral	0.02 g/kg	Alleviated morphological destruction of intestinal villi; Enhanced serum antioxidant capacity	[142]

Notes: IgA, immunoglobulin A; IgG, immunoglobulin G; IgM, immunoglobulin M; FITC-Dextran, fluorescein isothiocyanate-dextran; ADFI, average daily feed intake; FCR, feed conversion ratio.

## Data Availability

No new data were created or analyzed in this study.
